# Comparison of Results from the BARCODE1 Study and Contemporary Prostate Cancer Screening Trials

**DOI:** 10.1016/j.euo.2025.12.013

**Published:** 2025-12-31

**Authors:** Amit Sud, Alan McNeill, Andrew J. Vickers

**Affiliations:** a Department of Medical Oncology, Dana-Farber Cancer Institute, Boston, MA, USA;; b Broad Institute of MIT and Harvard, Cambridge, MA, USA;; c Harvard Medical School, Boston, MA, USA;; d Centre for Immuno-Oncology, Nuffield Department of Medicine, University of Oxford, Oxford, UK;; e Department of Urology, Western General Hospital, Edinburgh, UK;; f Department of Epidemiology and Biostatistics, Memorial Sloan Kettering Cancer Center, New York, NY, USA

**Keywords:** Prostate cancer, Screening, Polygenic risk score

## Abstract

Polygenic risk scores (PRSs) have generated considerable interest as a means of personalizing prostate cancer (PC) screening by stratifying individuals according to their genetic risk. The single-arm BARCODE1 study recently showed that PRS-based screening detected more PCs than prostate-specific antigen (PSA) testing or magnetic resonance imaging (MRI). The absence of a comparator arm limits the interpretability of this finding. We compared outcomes from BARCODE1 with those from two contemporaneous, large-scale screening trials—Göteborg-2 and ProScreen—that used MRI with or without a PC blood marker to risk-stratify men for biopsy. When standardized to 10 000 men tested, BARCODE1 biopsied more men (704 vs 386 and 338), diagnosed more low-grade PCs (126 vs 103 and 41), and detected fewer high-grade PCs (155 vs 178 and 165) versus Göteborg-2 and ProScreen. Combining PRS with PSA or MRI in BARCODE1 reduced the detection of high-grade PCs by 50–75% in comparison to Göteborg-2 and ProScreen. These findings reflect the limited risk discrimination of PRSs and their inability, unlike MRI and blood-based markers, to preferentially detect aggressive disease. PRS-based PC screening underperforms relative to current best practice and, on the basis of BARCODE1 data, should not be adopted in clinical practice.

Screening for prostate cancer (PC) using prostate-specific antigen (PSA) alone is of questionable net benefit because of the relatively high number of men that need to be biopsied and diagnosed to prevent one PC-specific death [1]. This has led to efforts to risk-stratify PSA screening to improve the ratio of benefits to harms.

Polygenic risk scores (PRSs) have generated considerable interest as a possible means of personalizing PC screening by stratifying individuals according to their genetic risk. A PRS combines information for multiple common genetic variants, each of which contributes a small amount of risk, to estimate an individual’s overall genetic predisposition to developing PC. Advocates propose that PRS-guided screening could better balance the benefits and harms of screening by targeting higher-risk individuals. McHugh et al [2] recently investigated this approach in the BARCODE1 study, a single-group study conducted in the UK in which all men aged 55–69 yr from 69 primary care practices were invited to participate. Volunteers underwent PRS testing, with those in the top 10% of the risk distribution invited for PSA measurement, magnetic resonance imaging (MRI), and biopsy. A biopsy was offered to all men at high PRS risk irrespective of PSA and imaging findings. In cases in which MRI-visible lesions were present, additional targeted biopsy cores were obtained. The authors claimed that the percentage of men “found to have clinically significant disease [on PRS-based screening] was higher than the percentage that would have been identified with the use of PSA or MRI.” [2]. Such a conclusion is questionable in the absence of a comparator arm.

To contextualize the performance of PRS-based screening, we compared the outcomes from BARCODE1 with those from two contemporaneous large-scale screening trials—Göteborg-2 and ProScreen [3,4]. Göteborg-2 is a randomized, population-based screening trial in Sweden [3]. Men aged 50–60 yr undergo repeated PSA testing, and those with PSA ≥ 3.0 ng/ml proceed to prostate MRI. Participants are then randomized to either systematic biopsy with additional targeted biopsy if MRI-visible lesions are present, or MRI-targeted biopsy alone. ProScreen is a randomized, population-based screening trial in Finland, enrolling men aged 50–63 yr [4]. In the intervention arm, men undergo PSA testing, and those with PSA ≥ 3.0 ng/ml receive further risk stratification using a PC blood marker, a four-kallikrein panel. Men with a kallikrein score ≥ 7.5% proceed to MRI, followed by targeted biopsy if indicated.

From each trial, we extracted data on the number of men screened and biopsied, and the number of high-grade (defined as Gleason ≥ 7, grade group ≥ 2) and low-grade (Glea-son 6, grade group 1) PCs detected. To facilitate comparison across studies, outcomes were standardized and are reported per 10 000 screened individuals. For example, in BARCODE1, 704 biopsies were performed per 10 000 menscreened (468 ÷ 6644 × 10 000), and detected 155 high-grade cancers (103 ÷ 6644 × 10 000) and 126 low-grade cancers (84 ÷ 6644 × 10 000). Raw numbers are shown here only to illustrate the calculation; all the results are presented in Table 1 as standardized outcomes per 10 000 screened.

A good screening strategy should maximize the yield of high-grade cancers detected but minimize the number of men biopsied and the number of low-grade cancers detected, which are generally considered to represent over-diagnosis. In comparison to screening strategies that are based on PSA and MRI (Göteborg-2) or PSA, markers, and MRI (ProScreen), the PRS-based screening strategy (BARCODE1) resulted in more men being biopsied, and more overdiagnoses of low-grade PC, yet detected fewer cases of high-grade disease per 10 000 men screened (Table 1). A PRS strategy combined with PSA or MRI—with men biopsied only if they have a high PRS plus high PSA, a positive MRI, or both—results in a very low yield of high-grade cancers, missing more than half of the cancers detected by other contemporary screening approaches.

As BARCODE1 did not include a control arm, placing its findings in context requires comparison with other contemporary screening trials. Inevitably, these trials differ in terms of patient characteristics and health care setting, all of which can influence outcomes. However, the differences in the numbers of biopsies, overdiagnoses, and cancers between PRS and other approaches are consistent and large, and it is unlikely that these could be explained by between-trial differences in patient characteristics. It is also notable that BARCODE1 screened an older population (55–69 yr) than Göteborg-2 (50–60 yr) and ProScreen (50–63 yr). Although higher incidence of clinically significant PC would be expected in this older cohort, BARCODE1 detected fewer high-grade cancers per 10 000 men screened.

The poor performance of PRS-based screening is perhaps unsurprising given its modest diagnostic properties. A central limitation is the weak risk discrimination offered by PRS, reflected both in the modest stratification of 10-yr absolute risk across PRS percentiles in BARCODE1 and in a low area under the receiver operating characteristic curve (AUC) of 0.67 [5,6]. To place the AUC in a clinically meaningful context, it is necessary to derive related performance metrics, specifically the detection rate (DR, or sensitivity; ie, the proportion of individuals with the disease who are correctly identified by the test) and the false positive rate (FPR; proportion of individuals without the disease who are incorrectly classified as high risk) [5]. Using the risk-screening converter, this AUC corresponds to a DR of 24% and FPR of 9% at the 90th percentile threshold (Fig. 1) [7]. By contrast, population-based cancer screening tests such as fecal immunochemical testing for colorectal cancer (DR = 79%, FPR = 6%) and digital mammography for breast cancer (DR = 75%, FPR = 8%) offer substantially stronger performance [8,9]. Expressed differently, most PCs in the population occur in men below the 90th percentile of PRS risk and are missed by the BARCODE1 PRS approach, but would be detected by the screening strategies used in Göteborg-2 and ProScreen. Consequently, at the population level, the PRS as implemented in BARCODE1 cannot reliably identify which men would benefit from continued PSA-based screening, unlike baseline PSA measured at ages 40–60 yr [10].

Crucially, current PC PRSs do not preferentially detect aggressive disease [11]. The proportions of high-grade and low-grade cancers are similar across current PC PRS risk strata, so restricting screening to higher-risk groups defined by PRS will not improve the balance of screening benefits and harms. We have previously demonstrated mathematically that the net benefit of screening can only be improved if the relative proportion of aggressive to indolent disease increases with higher scores [11]. This is true for PSA, markers, and MRI, which explains why screening approaches based on these modalities have superior properties. Future research should explore whether PRSs calibrated against PC mortality, rather than incidence, offer better clinical utility. A recent study con-cluded that given the additional costs of genotyping and downstream management combined with limited risk discrimination, realistic implementation of PRSs is unlikely to be cost effective [12].

Given their modest discriminatory ability, lack of preferential detection of aggressive disease, inferior performance in comparison to established screening strategies, and limited cost effectiveness, current PC PRSs do not offer a favorable balance of benefits to harms. Thus, they should not be used for screening in clinical practice at this time. Substantial methodological advances, particularly in the development of PRSs calibrated to lethal disease endpoints, are required before their role in population screening can be reconsidered.

## Figures and Tables

**Fig. 1 –:**
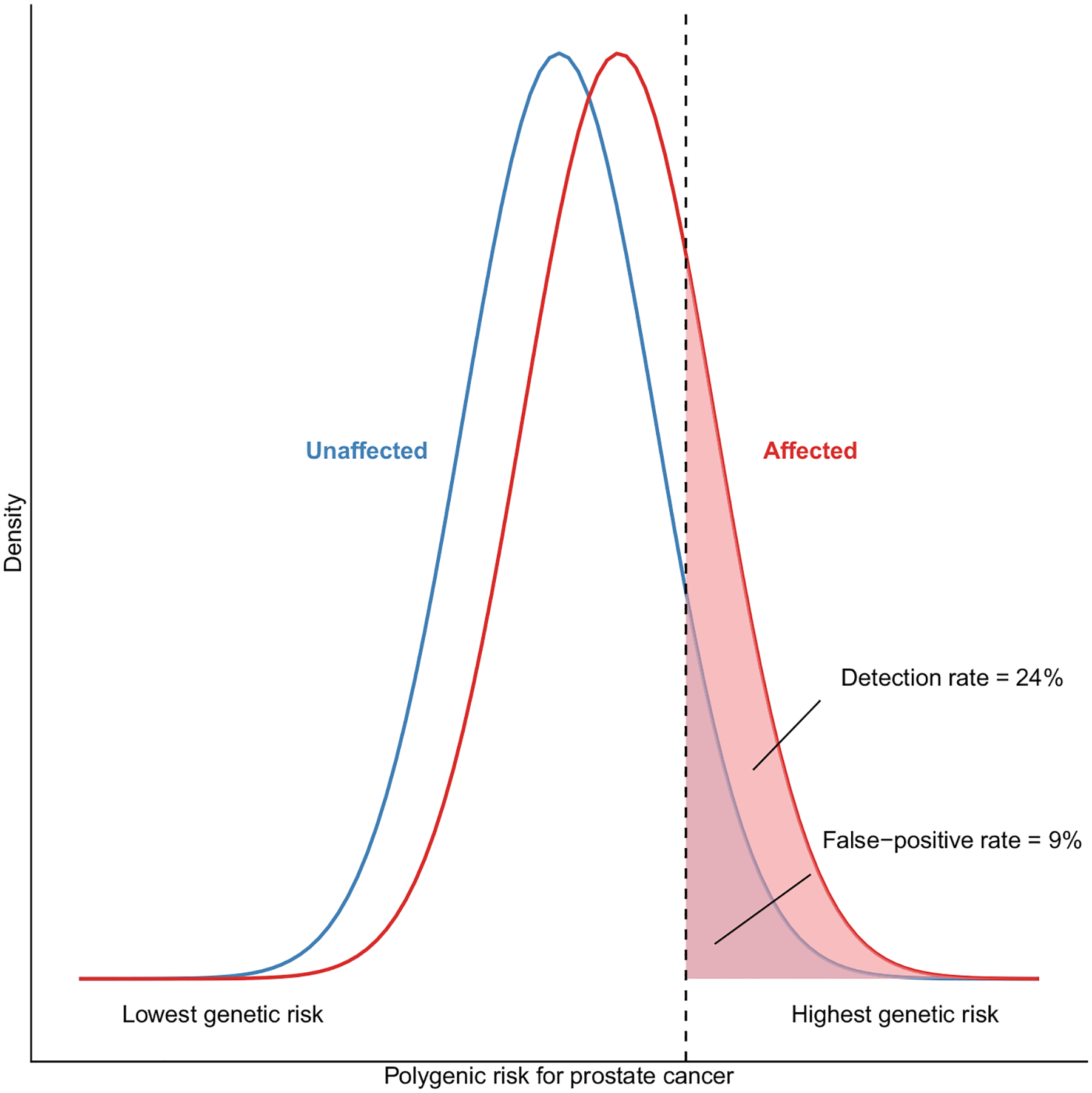
Density distributions of polygenic risk score (PRS) values for individuals with prostate cancer (red) and without prostate cancer (blue). Shaded regions correspond to a detection rate of 24% and a false positive rate of 9% according to a threshold at the 90th percentile of the PRS distribution. These performance metrics were derived from the area under the curve using the risk-screening converter [6,7].

**Table 1 –:** Comparison of outcomes for population-based prostate cancer screening approaches between the Göteborg-2, ProScreen, and BARCODE1 studies

Parameter^a^	Göteborg-2	ProScreen	BARCODE1			
Cohort age (yr)	50–60	50–63	55–69			
Screening modalities	PSA + MRI-TBx	PSA, 4KP, + MRI	PRS	PRS + PSA	PRS + MRI	PRS, PSA, + MRI
Participants biopsied	386	338	704	170	146	54
csPC cases diagnosed	178	165	155	78	68	42
ciPC cases diagnosed	103	41	126	26	24	3

PSA = prostate-specific antigen; MRI = magnetic resonance imaging; TBx = targeted biopsy; 4KP = four-kallikrein panel; PRS = polygenic risk score; csPC = clinically significant prostate cancer; ciPC = clinically insignificant prostate cancer.

a Numbers are expressed per 10 000 individuals screened to allow comparison across the screening approaches.

## ADVANCING PRACTICE

### What does this study add?

This is a comparative analysis of a polygenic risk score (PRS)-guided prostate cancer screening strategy (BARCODE1) versus two contemporary population trials that combine prostate-specific antigen, magnetic resonance imaging, and blood biomarkers (Göteborg-2 and ProScreen). We show that PRS-guided screening subjects more men to biopsy, detects fewer clinically significant cancers, and overdiagnoses more low-grade tumors than current best practice. Quantitative metrics highlight the limited risk discrimination of currently available PRSs. Given that these scores underperform relative to established methods and do not preferentially detect aggressive disease, they should not be adopted in clinical practice.

### Clinical Relevance

This comparative analysis demonstrates that polygenic risk score (PRS)-guided prostate cancer screening results in more biopsies, fewer clinically significant cancers detected, and increased overdiagnosis of low-grade tumors compared to established PSA- and MRI-based strategies. The findings highlight the limited discriminatory power of current PRS tools, which fail to preferentially identify aggressive disease, and underscore that PRS-based screening underperforms relative to contemporary best practices. Clinicians should continue to rely on validated PSA, biomarker, and MRI-based approaches for prostate cancer screening, as PRS implementation in its current form risks unnecessary procedures and missed high-risk cases. Associate Editor: Guillaume Ploussard.

### Patient Summary

We compared a genetic approach for prostate cancer screening (polygenic risk score) to two established methods that use MRI (magnetic resonance imaging) scans and blood tests for cancer markers. The genetic approach led to more biopsies but found fewer aggressive cancers and more low-risk tumors. Current polygenic risk scores for prostate cancer screening are less effective than standard methods such as measurement of PSA (prostate-specific antigen) and cancer markers and MRI scans.
